# Amplifying the Voices of Healthcare Workers in Conflict Settings: Comment on "Human Resources for Health in Conflict Affected Settings: A Scoping Review of Primary Peer Reviewed Publications 2016–2022"

**DOI:** 10.34172/ijhpm.9492

**Published:** 2026-03-31

**Authors:** Aula Abbara, Munzer Alkhalil

**Affiliations:** ^1^Department Infectious Diseases, Imperial College London, London, UK.; ^2^Syria Public Health Network, London, UK.; ^3^David Nott Foundation, London, UK.

**Keywords:** Conflict, Healthcare Workers, Human Resources, Attacks on Healthcare, Accountability

## Abstract

Attacks on healthcare are increasing globally, often with impunity and limited accountability with profound impacts on healthcare workers, the populations they serve and the wider health system, with effects that last well beyond the end of hostilities. Healthcare workers face impacts both on their personal and professional lives, with additional strains on their families which can lead them to emigrate, even where they may hold idealistic resolve to remain. This exodus (as well as the killing or detention) of their colleagues, places strains on the remaining healthcare workers and health system at a time when needs are at their highest. The cessation of such attacks, the naming of perpetrators and enforcing legal accountability are essential. To mitigate the long-term impacts on the health system, policies which build resilience into the production, distribution, retention, and demand components of health labour market (HLM) dynamics must be implemented in ways which are contextualised and dynamic.

 Armed conflicts are increasing globally, both in their complexity and impacts. Alongside, the changing geopolitical landscape, the widespread disregard for International Humanitarian Law, the normalisation of attacks on healthcare and the interruption of humanitarian services are having profound impacts on the delivery of services to vulnerable populations.^1^ Of grave concern is the limited accountability and prosecutions meted out to the perpetrators of these war crimes though there are current, ongoing inquiries. At the hard edge of these conflicts are healthcare and humanitarian workers who bear a personal and professional burden. The lack of accountability and disregard of legal norms to support them, both as civilians and professionals, together with weakened governance has profound impacts on their personal and professional lives, on retention and ultimately on post-conflict recovery. As such, Onvlee and colleagues’ review is a timely exploration of available literature on healthcare workers in conflict-affected settings through a Health Labour Market (HLM) framework lens.^2^ We draw on themes from this review to expand the discourse, emphasising the importance of centring the voices of those directly affected, examining power dynamics and inequalities related to gender and different cadres and exploring the impacts of attacks on healthcare for those left behind. We end by exploring policies which can support HLM resilience in these settings.

 Healthcare workers themselves are both “civilians” and “professionals” in settings of conflict; this means they face impacts both common to the wider community but with additional challenges. They may face persecution or attacks due to their role or leadership in a conflict setting either by the authorities eg, criminalisation of healthcare provision in Syria by the former Syrian regime or due to wider breakdowns in governance and accountability where they may be targeted by conflict actors or affected populations.^3^ This places strains on them and their families, particularly when conflict intensity is high, when healthcare workers are being disproportionately targeted or when their families are threatened. In some instances, they may choose a place of relative safety for their families (within or outside of their country borders) while they work in more extreme settings, leading to prolonged periods of uncertainty, security risks or separation. This can contribute to a mismatch in population needs versus the distribution of healthcare workers and negatively impact retention at a time where demand for healthcare workers is at its highest.

 From previous work on Syria, funded by Elrha’s R2HC (Research for Health in Humanitarian Crises) and led by Dr. Rohini Haar and Professor Leonard Rubenstein of the Safeguarding Health Coalition, we understand—through the voices of affected healthcare workers themselves—the impacts of attacks on both their personal and professional lives.^4^ Of note, are the profound impacts of “double tap” attacks, where one attack is a harbinger to a second attack, a tactic used by Russia to maximise casualties (including of health personnel and first responders) and interruption to the health facility.^4^ Similar tactics have been used by Russia in Ukraine and by Israel in Gaza.

 Additional stressors for healthcare workers occur when those left behind—either through the killing, detention or forced displacement of their colleagues—must work in health systems which are attacked or damaged, severely understaffed, severely under-resourced, overstretched and where they are unable to meet the needs of the populations served. This can exacerbate hopelessness and burnout and in themselves, negatively impact retention. A stark example of this is Gaza, where Israeli attacks have caused widespread damage to healthcare. Insecurity insight report more than 2001 incidents of violence against or obstruction of healthcare, the killing of 695 healthcare workers, arrest of 357 and damage to 34 out of 36 of Gaza’s hospitals(as of August 2, 2025).^5,6^ Such attacks occur at a time when population needs are at their most acute and complex with high demands for healthcare workers. The impact of such attacks, together with impunity causes extreme and often existential suffering, particularly when healthcare workers themselves have experienced the direct impacts of attacks on hospitals and their aftermath leading to strains on their mental health.

 The toll on the healthcare workers’ psychology is extreme and yet support tailored to their specific needs, remains inadequate. In Gaza, even before the current conflict, studies from 2014 showed levels of post-traumatic stress disorder to affect 89.3% of doctors and nurses.^7^ In Eastern Ghouta, an area in Syria which faced extreme besiegement by the former Syrian regime between 2013 and 2018, healthcare workers faced both extreme psychological distress alongside severe malnutrition and exhaustion.^8^ Beyond this, the psychological toll of making challenging ethical decisions with sparse resources or working beyond their training compounded their situation with one doctor saying: “The worst impact of the siege on doctors was the psychological burden of making life-or-death decisions due to the rationing of medicines and the unavailability of essential medical supplies. At one point, we had to ration the use of fluids, forcing doctors to make agonising choices about which patients would receive the limited treatment available.”^8^

## Centring Healthcare Worker Perspectives

 From the perspective of healthcare workers, insecurity, poor remuneration, unequal opportunities for training or employment, unaccredited degrees or unrecognised training (for those graduating from non-accredited universities outside of state control) can lead to significant demotivation, distress and weaken the resolve to remain, particularly where conflicts are protracted and severe.^2,4^ Previous research among Syrian healthcare workers has suggested that graduates with accredited degrees or training, physicians with recognised speciality certificates, those who hold travel permits, those with connections outside of their country or favourable destination countries policies are more able and likely to pursue careers abroad.^4,8^ The more protracted a conflict, the more likely the resolve of healthcare professionals to remain in an area of conflict breaks, particularly where future options are limited despite altruistic or idealist reasons they may have held to remain. This in part relates to their own aspirations but, from interviews conducted with affected healthcare workers, many state their decision to leave is related to their families and children, for reasons of safety, educational opportunities, and their futures.^4,8^

 Social sciences literature which explores the experiences of healthcare workers in conflict settings continues to expand though its scope, as seen in the Onvlee and colleagues’ review,^2^ does not equitably represent the voices of all cadres nor of all conflict-affected settings. We note that literature on Syria accounted for 11 of 36 included papers followed by the Democratic Republic of the Congo (n = 6), speaking to the strength of interest and of research in the Syrian context. Researching the Impact of Attacks on Healthcare,^9^ a UK government funded interdisciplinary research collaboration based at the University of Manchester has contributed to high quality research to this field with recent publications on Myanmar,^10^ Central African Republic,^11^ and Nepal.^12^ However, research from the perspectives of affected healthcare workers remains inadequate compared to the extent, particularly research which is participatory or co-designed between researchers and affected healthcare workers to fully contextualise the diversity of experiences.

 Based on Onvlee and colleagues’ review and other published work, we posit that the voices of non-physician cadres and the experiences of female healthcare workers (which may intersect,) are often under-explored. Though some research has attempted to address this gap,^13^ understanding the often-intersectional challenges faced by these groups is important as they face additional stressors. For example, social or familial pressures may impact decisions about work and careers such as distance or timing of travel, insecurity, access through checkpoints, the need to be escorted, being resident on-call away from home or holding responsibilities for child or elder care.^4^

## Impacts on the Production of Healthcare Workers

 The production of healthcare workers whether by universities or institutes and their postgraduate training is often negatively impacted by attacks on healthcare. Direct attacks on universities have occurred in Sudan, Syria, Gaza, Myanmar, and Ukraine; to compound this, the killing or forced exodus of senior educators have both immediate and long-term consequences on the healthcare workforce both for production and quality. This can negatively impact the early recovery or health system rebuilding phase in a post-conflict setting. For physicians, this means that though they continue to be graduated, perhaps contributing to an overproduction in some areas or specialties, the interruptions to governance and workforce planning may result in a mismatch between production and demand. For example, psychiatry, an already neglected speciality in many conflict settings may be further deprioritised despite rising needs among the population. In conflict and post-conflict settings, rehabilitation physicians and allied healthcare workers eg, physiotherapists, prosthetists may be in high demand but in short supply, negatively impacting care for vulnerable patients.

 The training needs of physicians and their role within a health system are often more complex compared to other cadres. For example, training for specialist surgeons may take several years of postgraduate training, mentorship, examinations and require specialised equipment. The collapse of postgraduate training for physicians in conflict can therefore have significant implications.^14^ However, the production and training of non-physician cadres may be less emphasised during conflict though the role of nurses, midwives, pharmacists, “technicians” and community healthcare workers are no less important than that of physicians. Policies which reimagine training and support for these cadres in conflict settings is essential as, though there have been examples of good practice, these cadres may be neglected. This may include opportunities for training, taking on more responsibilities with associated renumeration or opportunities for task sharing. Developing new cadres to meet essential gaps may also be needed. For example, in Syria, “technicians” have been supported to take on specialist roles; their training includes “on-the-job” training, often as often as an apprenticeship for two to three years, allowing them to work in various specialisms including laboratories, anaesthetics, renal dialysis, intensive care or in anaesthetics. As such, they fill an important gap in the healthcare workforce, working under the supervision of a physician or specialist nurse at a time when other cadres are in short supply.

## Building Resilience in Production and Demand

 The factors discussed contribute to mismatched production and demand of healthcare workers with long term impacts on the health system and ultimately on population health. HLM dynamics are closely related to both conflict dynamics and the pre-conflict state of a country and its workforce governance as these impact the production, training and retention of healthcare workers. In complex or protracted conflicts—or when conflicts are dynamic and vary in acuity over time or geography—population needs may also evolve, requiring different cadres, specialities, distributions, or quantities of healthcare workers.

 Even in non-conflict or stable settings, HLM planning is challenging. In conflict settings, matching production and demand presents additional challenges and requires dynamic use of the HLM framework and the introduction of realistic policies which recognise evolving local governance structures, their capacity, legitimacy and the projected needs of the (sub)populations in different geographical areas. Such dynamics also have implications in the post-conflict period when policies and incentives to support healthcare workers to return need to be attractive enough to mitigate ongoing insecurity, limited remuneration or challenges to professional development. For this to be effective, accountability and transitional justice with clear governance structures to support and retain the healthcare workforce are essential.

 Building resilience into HLM forces in a protracted conflict must focus not only on production and demand but also retention and equitable distribution. Policies which protect and diversify training pipelines, recognise qualifications earned outside formal governmental jurisdiction, consider bonded scholarships and hazard pay retainers for underserved locations, and which support continuous professional development can support distribution and retention. On the demand side, humanitarian health aid alignment with national needs and strategies, enhancing aid through recipient governments, increasing pooled funds, and shifting to multi-year financing would create demand in the right places to support universal health coverage. At the local level, policies which stipulate transparent posting and transfer rules, and which offer bundle incentives that combine salary with safety measures, housing, and career progression, especially for women and non-physician cadres can play a role in improving the retention of health workers and supporting equitable distribution.^15^

 A particular challenge to HLM planning in conflict settings is gathering sufficiently accurate data on the healthcare workforce (including cadres, work patterns, seniority, dual practice, distribution), vacancy rates and on population needs. Though remuneration is important, policies which support the working lives of healthcare workers—particularly women—can support retention, even in the most dire of conflicts with many choosing to remain through a sense of duty as seen in Gaza, Ukraine, Syria, Lebanon, Iran, Sudan, and other countries. These may include policies around maternity leave, support for breast feeding, affordable on-site childcare and flexible hours eg, part-time hours to support women in the workforce. For non-physicians, policies which support career progression and training opportunities associated with seniority and renumeration can support retention.

## Conclusion

 We make clear that attacks on healthcare and healthcare workers must cease, perpetrators must be held accountable and United NationsSecurity Council Resolution 2286 (May 2016) on “healthcare in armed conflict” must be upheld; those responsible must be held to account. The impact on healthcare workers of conflict as both civilians and professionals is grave and their loss to the health system has long term impacts on the health system and population health. Policies which support the working lives and career progression of healthcare workers (particularly women and non-physician cadres) can support retention, particularly where conflicts are protracted. These must be implemented alongside policies which build resilience into HLM dynamics which are contextualised and responsive to conflict dynamics.

## Disclosure of artificial intelligence (AI) use

 Not applicable.

## Ethical issues

 Not applicable.

## Conflicts of interest

 Authors declare that they have no conflicts of interest.
